# Supplementary figures and images for: Ancient DNA Reveals Maternal Philopatry of the Northeast Eurasian Brown Bear (Ursus arctos) Population during the Holocene
Source: Genes (Basel). 2022 Oct 27;13(11):1961. doi: 10.3390/genes13111961 (PMC9689912; doi:10.3390/genes13111961)

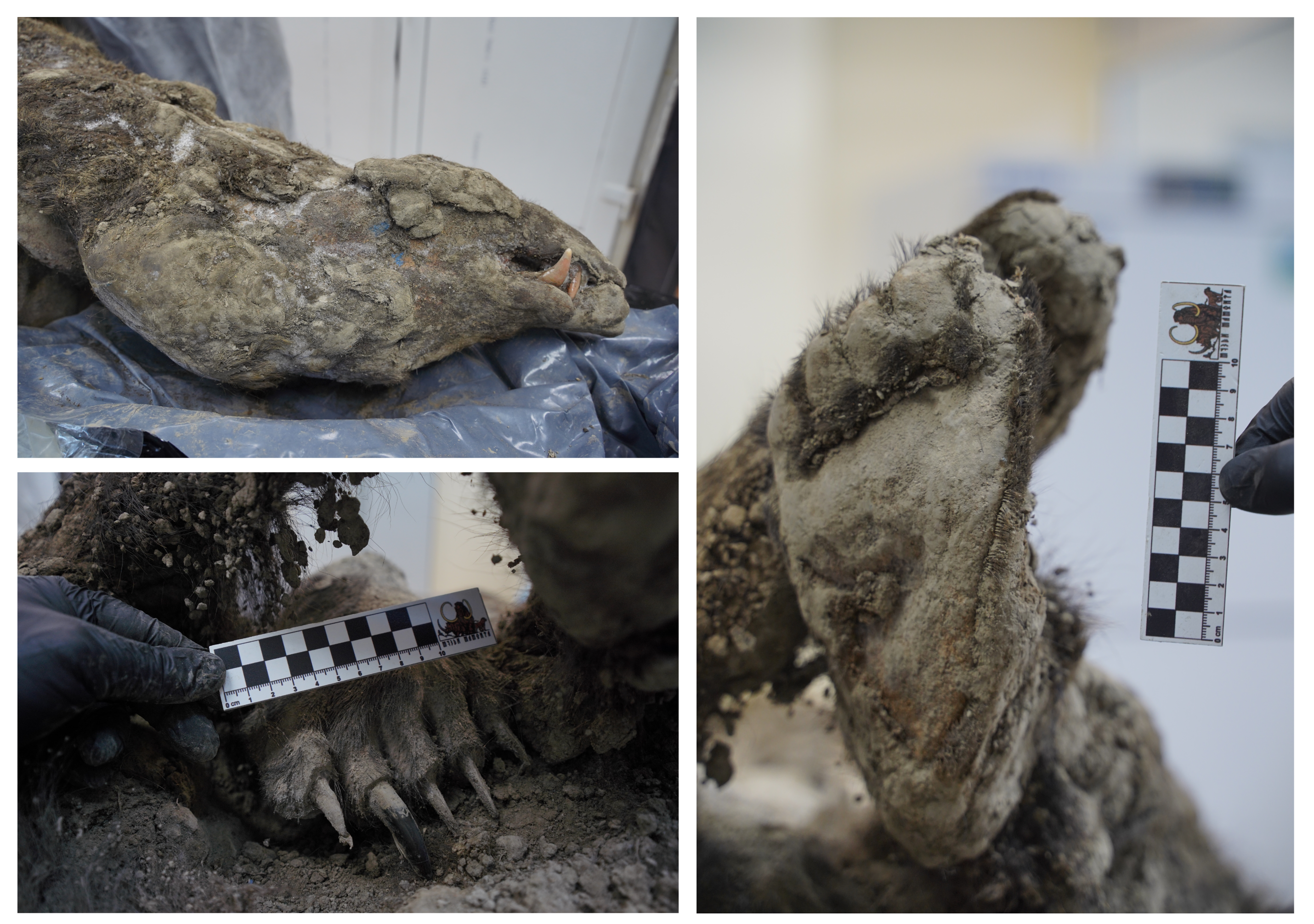

Supplement: Supplementary file 1 [file genes-13-01961-s001.zip › supplementary_file/Figure_S1.png]

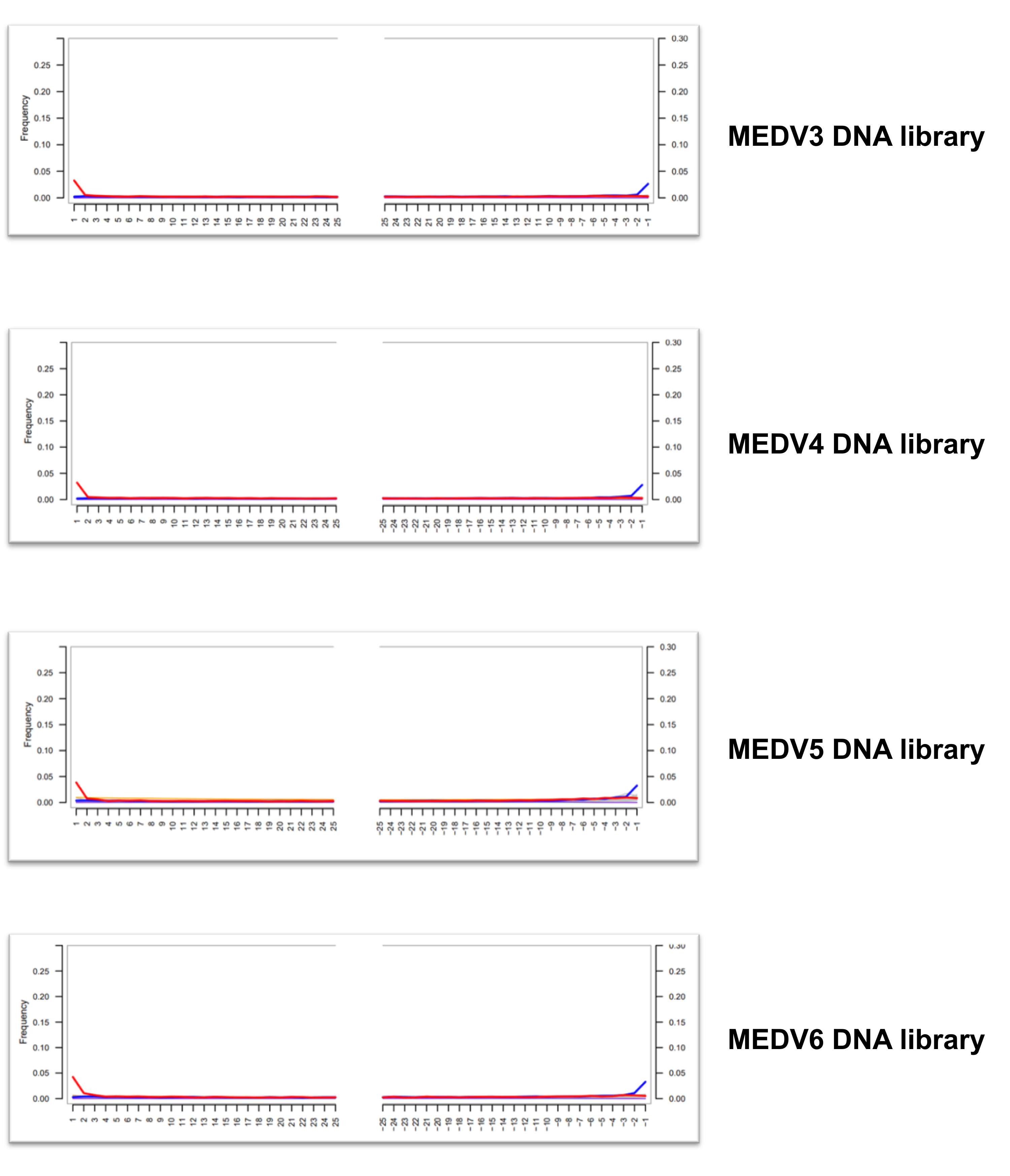

Supplement: Supplementary file 1 [file genes-13-01961-s001.zip › supplementary_file/Figure_S2.png]

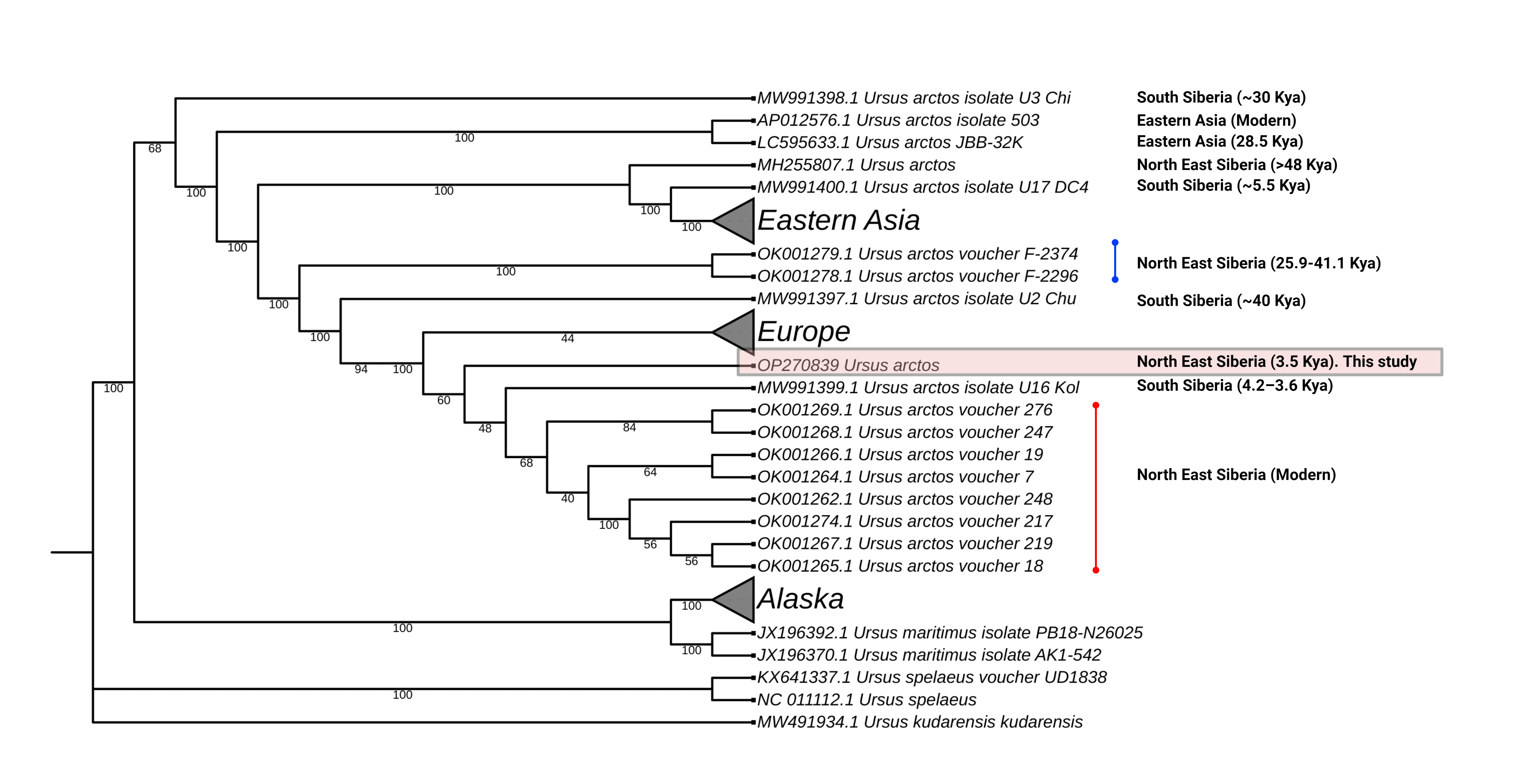

Supplement: Supplementary file 1 [file genes-13-01961-s001.zip › supplementary_file/Figure_S3.png]
